# The cell nuclei of skeletal muscle cells are transcriptionally active in hibernating edible dormice

**DOI:** 10.1186/1471-2121-10-19

**Published:** 2009-03-14

**Authors:** Manuela Malatesta, Federica Perdoni, Serafina Battistelli, Sylviane Muller, Carlo Zancanaro

**Affiliations:** 1 Dipartimento di Scienze Morfologico-Biomediche, Sezione di Anatomia e Istologia, University of Verona, Italy; 2 Dipartimento di Biologia Animale, Laboratorio di Biologia Cellulare, University of Pavia, Italy; 3 Istituto di Istologia e Analisi di Laboratorio, University of Urbino, Italy; 4 CNRS, Institut de Biologie Moléculaire et Cellulaire, Immunologie et Chimie Thérapeutiques, Strasbourg, France

## Abstract

**Background:**

Skeletal muscle is able to react in a rapid, dynamic way to metabolic and mechanical stimuli. In particular, exposure to either prolonged starvation or disuse results in muscle atrophy. At variance, in hibernating animals muscle atrophy may be scarce or absent after bouts of hibernation i.e., periods of prolonged (months) inactivity and food deprivation, and muscle function is fully preserved at arousal. In this study, myocytes from the quadriceps muscle of euthermic and hibernating edible dormice were investigated by a combination of morphological, morphometrical and immunocytochemical analyses at the light and electron microscopy level. The focus was on cell nuclei and mitochondria, which are highly sensitive markers of changing metabolic rate.

**Results:**

Findings presented herein demonstrate that: 1) the general histology of the muscle, inclusive of muscle fibre shape and size, and the ratio of fast and slow fibre types are not affected by hibernation; 2) the fine structure of cytoplasmic and nuclear constituents is similar in euthermia and hibernation but for lipid droplets, which accumulate during lethargy; 3) during hibernation, mitochondria are larger in size with longer cristae, and 4) myonuclei maintain the same amount and distribution of transcripts and transcription factors as in euthermia.

**Conclusion:**

In this study we demonstrate that skeletal muscle cells of the hibernating edible dormouse maintain their structural and functional integrity in full, even after months in the nest. A twofold explanation for that is envisaged: 1) the maintenance, during hibernation, of low-rate nuclear and mitochondrial activity counterbalancing myofibre wasting, 2) the intensive muscle stimulation (shivering) during periodic arousals in the nest, which would mimic physical exercise. These two factors would prevent muscle atrophy usually occurring in mammals after prolonged starvation and/or inactivity as a consequence of prevailing catabolism. Understanding the mechanisms responsible for skeletal muscle preservation in hibernators could pave the way to prevention and treatment of muscle wasting associated with pathological conditions or ageing as well as life in extreme environments, such as ocean deeps or spaceflights.

## Background

Skeletal muscle is able to rapidly respond to mechanical as well as metabolic stimuli such as food intake and changes in the frequency and duration of use. In particular, prolonged starvation and disuse normally lead to muscle atrophy. Starvation activates intense lipolysis and proteolysis in several tissues inclusive of skeletal muscle, with increased muscle protein breakdown and final loss of muscle mass (reviewed in [1]); disuse induces muscle atrophy characterised by loss of muscle mass, decreased cross-sectional area of muscle fibre and lower amounts of contractile protein – especially myosin heavy-chain proteins – mainly due to the unbalanced regulation of protein catabolism and anabolism [2,3]. This phenomenon affects skeletal muscle after prolonged inactivity, whether by immobilization, spaceflight, spinal cord isolation, or denervation, although with variable effects depending upon muscle type and fibre type (e.g. [4-7]).

Interestingly, hibernators may show reduced or absent muscle atrophy as well as preservation of muscle function (e.g. [8-13]) after bouts of hibernation i.e., periods of prolonged inactivity and food deprivation lasting a few days to several months. In fact, these animals have evolved the capacity of entering a state of torpor characterized by a drastic reduction of body temperature, metabolic and physiological activities, and energy demand as well, thus overcoming adverse environmental conditions [14]. Therefore, hibernators provide a fascinating model for investigating muscle atrophy associated with disuse.

Most studies on skeletal muscle in hibernating animals deal with the histology, physiology or biochemistry of muscle tissue with scarce attention paid to the morpho-functional features of muscle cell constituents. In this study, we used a panel of morphological, morphometrical and immunocytochemical techniques to investigate, at the light and electron microscopy level, myocytes of the quadriceps muscle from euthermic and hibernating edible dormice; in this paper, results obtained in the cell nucleus and the mitochondria, which are highly sensitive to changing metabolic rate, are presented.

## Methods

### Animals and tissue processing

Six male edible dormice *Glis glis *(Gliridae) were used in this study. This dormouse is a common wild species in Italy and a limited number of individuals could be captured, for the purpose of multiple investigations, under permission from Authorities (Decreto n. 76 del 20 Gennaio 1998, Regione del Veneto); the experimental research was performed following internationally recognized guidelines. Adult (approximately 1–2 year old) wild-living animals were trapped and maintained in an outdoor animal house supplied with food and bedding material. Under such conditions, they spontaneously began to hibernate in November and awoke in March. Three animals were killed during the euthermic period (June-July) and three during deep hibernation (January). Euthermic animals were anesthetized with ether before decapitation; hibernating animals were taken from the cage and immediately killed.

Samples of the right quadriceps muscle were processed for transmission electron microscopy for either conventional morphology or immunocytochemistry. For morphological analysis, muscle samples were fixed by immersion in 2.5% (v/v) glutaraldehyde and 2% (v/v) paraformaldehyde in 0.1 M Sörensen phosphate buffer pH 7.4 at 4°C for 2 h, post-fixed with 1% (v/v) OsO_4 _at 4°C for 1 h, dehydrated through graded acetones and embedded in Epon 812. For immunocytochemistry, muscle samples were fixed by immersion in 4% (v/v) paraformaldehyde in 0.1 M Sörensen phosphate buffer at 4°C for 2 h, washed, treated with NH4Cl 0.5 M in PBS 0.1 M pH 7.4 to block free aldehydes, dehydrated with ethanol and embedded in LRWhite resin polymerized under U.V. light.

### Light microscopy

Transverse, two-μm-thick sections of LRWhite embedded muscle samples were either stained with toluidine blue or submitted to immunohistochemical procedures for fibre typing [15]. Sections were incubated for 2 h at room temperature with a mouse monoclonal antibody recognizing the heavy chain of skeletal fast fibre myosin (clone MY-32, Sigma-Aldrich, Buchs, Switzerland); the antigen-antibody complex was revealed with an Alexa 488 conjugated antibody against mouse IgG (Molecular Probes, Invitrogen., Milan, Italy). The sections were finally counterstained for DNA with 0.1 μg/ml Hoechst 33258, in order to detect apoptotic nuclei on the basis of chromatin morphology. Micrographs were taken with an Olympus BX51 microscope equipped with a 100 W mercury lamp under the following conditions: 330- to 385-nm excitation filter (excf), 400-nm dichroic mirror (dm), and 420-nm barrier filter (bf), for Hoechst 33258; 450- to 480-nm excf, 500-nm dm, and 515-nm bf for Alexa 488. Images were recorded with an Olympus Camedia C-5050 digital camera, and stored on a PC by the Olympus software for processing and printing.

Morphometrical evaluation of fibre size was performed on samples immunolabelled for fast myosin (to assess fast and slow fibre size separately): the cross-sectional area of 100 muscle fibres per sample was measured at 40× using the Image J software (NIH). Moreover, the percentage of fast and slow muscle fibres was calculated on a total of 400 fibres per animal.

Finally, the percentage of apoptotic myonuclei over a total of 1,000 cell nuclei per animal was calculated.

### Transmission electron microscopy

Ultrathin sections were placed on grids coated with a Formvar-carbon layer. Epon-embedded sections were stained with uranyl acetate and lead citrate prior to observation; LRWhite-embedded sections were processed for immunocytochemistry as follows. Sections were treated with either monoclonal antibodies directed against the phosphorylated form of polymerase II (Research Diagnostics Inc., Flanders, NJ, USA) or polyclonal antibodies directed against DNA/RNA hybrid molecules [16] specifically occurring in the transcriptional sites [17]. Sections were floated for 3 min on normal goat serum (NGS) diluted 1:100 in PBS, incubated for 17 h at 4°C with the primary antibody diluted in PBS containing 0.1% (w/v) bovine serum albumin (Fluka, Buchs, Switzerland) and 0.05% (v/v) Tween 20. After rinsing, sections were floated on NGS, and then reacted for 20 min at room temperature with the secondary 12 nm gold-conjugated antibody (Jackson ImmunoResearch Laboratories Inc., West Grove, PA, USA) diluted 1:10 in PBS. Finally, the sections were rinsed and air-dried. Control grids were treated as above but omitting the primary antibody from the incubation mixture, and then processed as described. In order to clearly identify the nuclear structural constituents containing ribonucleoproteins (RNPs), all the immunolabelled sections were further stained with the EDTA method [18].

All grids were observed in a Philips Morgagni TEM operating at 80 kV and equipped with a Megaview II camera for digital image acquisition. The gold grain contrast was digitally enhanced on micrographs by using Adobe Photoshop.

Morphometrical evaluations (×11,000) were made on 15 myonuclei per animal by using a computerized image analysis system (AnalySIS Image processing, Soft Imaging System GmbH, Muenster, Germany). The following parameters were considered: area of nuclei, nucleoli, and fibrillar centres (FCs); percentage of nuclear area occupied by condensed chromatin; percentage of dense fibrillar component (DFC) and granular component (GC) per nucleolus. Moreover, the sectional area as well as the inner and outer membrane profile length was measured (×22,000) in 40 mitochondria per animal; the inner-to-outer membrane length ratio was then calculated in order to estimate the inner membrane length independently from the mitochondrial size.

In order to obtain quantitative estimate of immunostaining, the labelling density over myonuclei was evaluated; sections from the same immunolabelling experiment were only scored. The surface area of 15 myonuclei per animal was measured (×22,000); gold grains were counted and the labelling density was expressed as the number of gold grains per square micrometer. For background evaluation, the resin outside the tissue was considered.

### Statistics

For each analyzed variable, the Kolmogorov-Smirnov two-sample test was performed in order to verify the hypothesis of identical distributions among animals of each group. The data for each variable were then pooled according to the experimental groups and the mean ± standard error of the mean (SE) was calculated. Statistical comparisons were performed by one-way ANOVA test (significance set at P ≤ 0.05).

## Results

### Light microscopy

**Figure 1:**
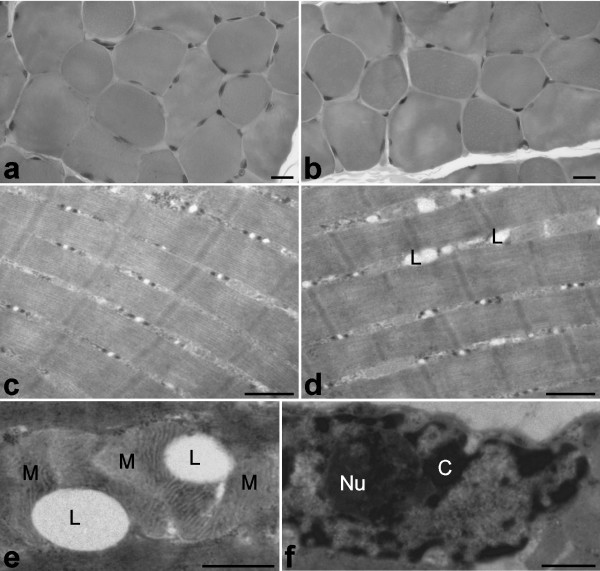
**Skeletal muscle cells from euthermic (a, c) and hibernating (b, d-f) edible dormice; Epon-embedded samples, uranyl acetate and lead citrate staining**. During hibernation, myofibres do not show evident structural modifications at both the light (a, b) and electron microscopic level (c, d) apart from the presence of some lipid droplets (L) (d), which are frequently surrounded by mitochondria (M) (e). Myonuclei also display their usual morphology with elongated shapes, finely irregular borders, condensed chromatin clumps (C) distributed both at the nuclear and nucleolar periphery, and one roundish nucleolus (Nu). Bars = a, b: 20 μm; c-f: 0.5 μm.

Morphological observation of muscle samples revealed similar features in euthermic and hibernating dormice (Fig. 1a, b); in particular, no evidence of necrosis or atrophy was observed in hibernating animals. Apoptotic myonuclei were quite scarce: they represented 0.33 ± 0.19% and 0.19 ± 0.13% of all myonuclei in euthermic and hibernating animals, respectively (P = 0.581).

**Figure 2:**
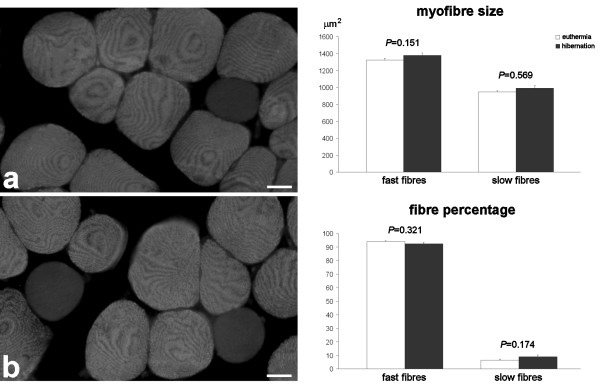
**Immunolabelled fast type II fibres in the quadriceps of euthermic (a) and hibernating (b) edible dormice, LRWhite-embedded samples**. No difference in size of both slow and fast myofibres, nor in percentage of fibre types occur in euthermic and hibernating dormice. Bars = 20 μm.

Morphometry showed that fast myofibres have significantly larger cross-sectional area than slow myofibres in all animals; however, myofibre cross-sectional area was similar in either fibre type during euthermia and hibernation, even when the data were expressed as percentage (Fig. 2).

### Transmission electron microscopy

**Figure 3:**
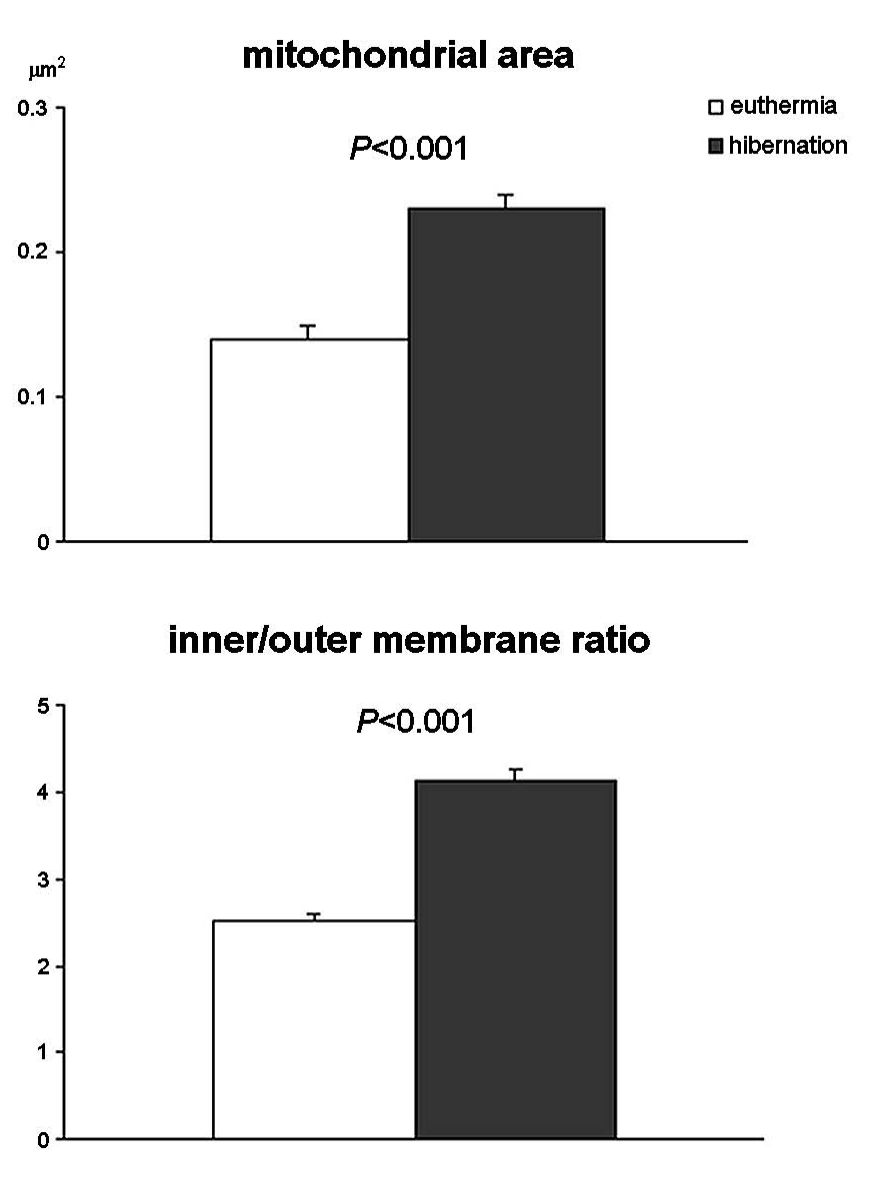
**Morphometry of mitochondria in muscle cells from euthermic and hibernating dormice**. Means ± SE.

**Figure 4:**
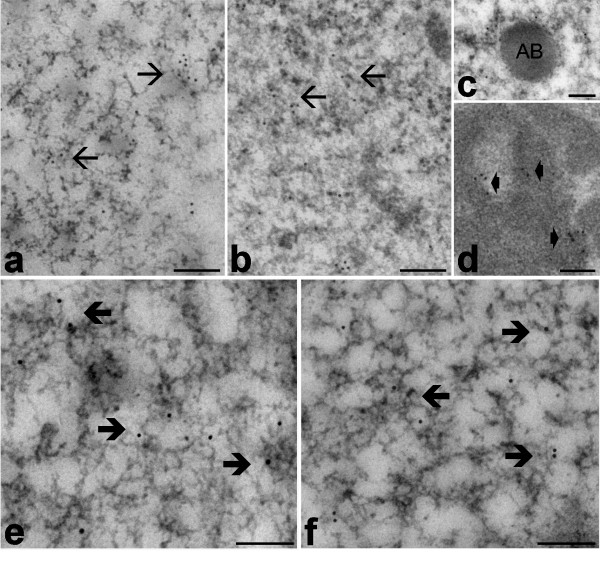
**Myonuclei from euthermic (a, e) and hibernating (b-d, f) edible dormice; LRWhite embedding, EDTA staining**. a-d. Immunolabelling with anti-DNA/RNA hybrid molecule antibody: the signal occurs in both nucleoplasm, where it is mainly associated to PF (arrows), and in nucleolar DFC (arrowheads). The amorphous body (AB) is unlabelled. e, f. Immunolabelling with anti-phosphorylated polymerase II antibody: a specific signal is present over PF (thick arrows). Bars = 0.2 μm.

In all dormice, skeletal muscle cells showed their typical, elongated shape with most of the cytoplasm occupied by the longitudinally arrayed myofibrils composed of thick (myosin) and thin (actin) filaments (Fig. 1c, d). The mitochondria were lined in small, longitudinally oriented cytoplasm areas between myofibrils; they were ovoid in shape with many transverse cristae in both euthermic and hibernating dormice; however, in mitochondria of hibernating animals both size and inner-to-outer membrane ratio significantly increased (Fig 3). Glycogen was more abundant in euthermia, especially close to mitochondria; on the other hand, during hibernation lipid droplets accumulated in the cytoplasm in close proximity to mitochondria (Fig. 1d, e). Multiple, elongated myonuclei occurred at the periphery of the cell, close to the plasma membrane: they generally showed finely irregular borders, condensed chromatin clumps at both the nuclear and nucleolar periphery, and one roundish nucleolus characterized by a few FC, and abundant DFC and GC (Fig. 1f). In the nucleoplasm, all the usual RNP structural constituents involved in pre-mRNA transcription and processing were clearly recognizable: perichromatin fibrils (PF) i.e. the in situ form of pre-mRNA transcription and early splicing [19]; perichromatin granules (PG), acting as both vector and storage site of already spliced pre-mRNA [19]; interchromatin granules (IG), sites for the storage and/or assembly of pre-splicing complexes [20,21]. In addition, amorphous bodies (Fig. 4c), a nuclear structural constituent found in different tissues during hibernation [22,23], were occasionally observed in myonuclei of hibernating dormice (at least one in about 2% of sectioned myonuclei). Morphometry (Table 1) showed that all the investigated structural parameters in myonuclei were similar in euthermic and hibernating dormice.

**Table 1:** Morphometry of myonuclei in euthermic and hibernating dormice.

	nuclear area (μm^2^)	chromatin %	nucleolar area (μm^2^)	FC area (μm^2^)	FC %	DFC %	GC %
Euthermia	11.56 ± 0.79	25.75 ± 0.94	1.84 ± 0.15	0.03 ± 0.002	2.77 ± 0.29	33.41 ± 1.44	64.16 ± 1.36
Hibernation	12.32 ± 0.71	26.52 ± 1.24	1.55 ± 0.14	0.04 ± 0.003	3.19 ± 0.38	38.13 ± 2.09	59.09 ± 2.11
P	0.481	0.625	0.455	0.178	0.697	0.354	0.316

Means ± SE.

In all samples, the antibody recognizing DNA/RNA hybrid molecules (an *in situ *marker of transcription) specifically labelled PF and the nucleolar DFC (Fig. 4a–d), whereas the anti-polymerase II signal was located on PF only (Fig. 4e, f). The labelling density (gold grains/μm^2^) of DNA/RNA hybrids was similar in euthermic and hibernating dormice (8.64 ± 0.35 vs 8.34 ± 0.24, P = 0.538); the same was found for phosphorylated polymerase II (8.99 ± 0.75 vs 9.46 ± 0.66, P = 0.656). The background labelling was negligible in all immunolabelling experiments (not shown).

## Discussion

Light and electron microscopic analysis of quadriceps muscle cells from euthermic and hibernating edible dormice demonstrated that: 1) the general structure of the muscle (histology, shape and size of myofibres, ratio of fast and slow fibre types) is not affected by hibernation; 2) the fine structure of both cytoplasmic and nuclear constituents is similar in euthermia and hibernation, with the exception of lipid droplets accumulating during lethargy; 3) during hibernation, mitochondria are larger in size with longer cristae, and 4) during hibernation, myonuclei maintain the same amount and distribution of transcripts and transcription factors as in euthermia.

Findings presented here demonstrate the absence of muscle atrophy and gross fibre changes in the edible dormice during hibernation, thereby confirming previous data in the ground squirrel [8,9], bear [10-12], and bat [13] showing that muscle mass, myofibre size or number, or the cell content in myofibrils are similar in skeletal muscle throughout the euthermia-hibernation cycle. A novel finding presented in this paper is that the myonucleus, a key organelle for most cellular activities, maintains an active ultrastructural configuration even after several months of immobility.

In hibernating edible dormice, mitochondria only show minor ultrastructural modifications, being larger and with longer cristae; similar changes are present in different tissues of hibernating hazel dormice [24], possibly due to preferential utilization of lipid as the source of energy during lethargy [14,25,26]. In fact, when fatty acids become the main substrate for respiration instead of carbohydrates, mitochondria increase their size and the number of cristae (see [24] and references therein). Consistently, the close association of muscle cell mitochondria with lipid droplets accumulating in the cytoplasm during hibernation (this study) parallels the increase in fatty acid binding proteins [27,28]; these changes are probably instrumental to efficient transport of fatty acids to the sites of utilisation. Therefore, the observed mitochondrial changes could help keeping respiration active under the extreme metabolic conditions of lethargy; in accordance, respiratory activity in skeletal muscle mitochondria was found to be similar in hibernating and euthermic ground squirrels [29]. Moreover, maintaining mitochondria in an "active" arrangement would allow hibernating animals to rapidly and fully restore mitochondrial function upon arousal i.e., a phase of exploding energy demand. This is of special relevance for skeletal muscle cells because all mammalian hibernators re-warm at periodic intervals during winter [30,31], shivering playing a key role in rising body temperature during arousal.

Previous studies on cell nuclei of different tissues from the edible and hazel dormice demonstrated quali-quantitative changes in PF and PG as well as architectural and molecular modifications of nucleoli (e.g. [22,32,33]); in addition, several different nuclear bodies involved in the storage/assembly of RNA processing factors have been shown to appear or increase in number during hibernation [23]. On the contrary, myonuclei of hibernating dormice exhibit an "active" appearance: in fact, RNP structural constituents involved in pre-mRNA processing (which are very sensitive to changes in transcriptional activity, see e.g. [34]), do not vary in their amount or distribution. Accordingly, the amorphous bodies, a typical nuclear body in hibernation [23], occur in the nucleoplasm of myonuclei in very limited amounts (2% of sectioned myonuclei showing one amorphous body vs 25% sectioned nuclei of hepatocytes presenting one to three amorphous bodies). Moreover, no change apparently occurs in the nucleolus, the site of rRNA transcription and ribosome assembly, which undergoes very rapid change in morphology and function according to changing cell metabolism (see e.g. [35]). During hibernation, myonuclei also maintain the same euthermic level of transcripts and transcription factors; this finding suggests either preservation of high functional rate in the nucleus or the establishment of a nuclear configuration able to quickly and massively restore the transcriptional activity where needed. The latter hypothesis is supported by evidence showing accumulation of phosphorylated polymerase II in hibernating ground squirrels in the presence of an overall decrease of transcriptional activity in skeletal muscle [36]. Accordingly, it has been recently reported [37,38] that mRNA transcripts accumulate during hibernation and quickly disappear at early arousal in various tissues of different hibernators, thus supporting the view that modulation of transcriptional activity is part of a far-reaching program leading to the rapid increase of protein synthesis upon arousal.

In skeletal muscle cells of hibernating mammals, the expression levels of major myofibrillar proteins are retained [13] or even up-regulated [39], so that protein synthesis and breakdown are balanced [40]. The current observations in the edible dormouse strongly support the concept that, even in deep hibernation, skeletal muscle cells maintain their synthetic activity at such a rate to effectively counteract muscle wasting (mind that degradation processes also slow down during hibernation) as well as support their ability to quickly restore in full the contractile function. The ongoing low-rate activity in skeletal muscle cells during hibernation could be related to low transmitter release at the neuromuscular junction, which has been found in hindlimb of the golden hamster [41], as well as to the trophic effect of enhanced purinergic activity [42]. In addition, the strenuous muscle shivering rapidly raising body temperature and metabolic rate at every periodic arousal could also help to prevent muscle atrophy by acting as intensive physical exercise [13,43].

## Conclusion

In this study we demonstrated that skeletal muscle cells of hibernating edible dormice maintain their structural and functional integrity in full, even after months of immobility. This would be due to maintenance, although at a low rate, of nuclear and mitochondrial activities, which would in turn counterbalance myofibre wasting during hibernation, as well as to the intensive stimuli coming from shivering during periodic arousal, which would mimic physical exercise.

These processes would prevent muscle atrophy typically occurring in mammals after prolonged starvation and/or inactivity as a consequence of the catabolic processes prevailing over the anabolic ones (e.g. [2-7]). It is worth mentioning that understanding the mechanisms responsible for skeletal muscle preservation in hibernators could pave the route to treatment and/or prevention of muscle wasting associated with pathological conditions or ageing as well as life in extreme environments, such as ocean deeps or spaceflights [44].

## Abbreviations

DFC: dense fibrillar component; FC: fibrillar centre; GC: granular component; IG: interchromatin granules; NGS: normal goat serum; PF: perichromatin fibrils; PG: perichromatin granules; RNP: ribonucleoprotein; SE: standard error of the mean.

## Authors' contributions

MM conceived the study, participated in sample processing, performed electron microscopy analyses and drafted the manuscript; FP carried out immunofluorescence analyses and morphometrical evaluations; SB participated in animal care, sample collection and processing; SM produced antibodies and helped to draft the manuscript; CZ participated in the design and coordination of the study and helped to draft the manuscript. All authors read and approved the final manuscript.
